# A Central Role for Atg5 in Microbiota-Dependent Foxp3^+^ RORγt^+^ Treg Cell Preservation to Maintain Intestinal Immune Homeostasis

**DOI:** 10.3389/fimmu.2021.705436

**Published:** 2021-08-26

**Authors:** Carlos Plaza-Sirvent, Bei Zhao, Alisha W. Bronietzki, Marina C. Pils, Neda Tafrishi, Marc Schuster, Till Strowig, Ingo Schmitz

**Affiliations:** ^1^Department of Molecular Immunology, Ruhr-University Bochum, Bochum, Germany; ^2^Systems-Oriented Immunology and Inflammation Research Group, Helmholtz Centre for Infection Research, Braunschweig, Germany; ^3^Institute of Molecular and Clinical Immunology, Otto-von-Guericke University, Magdeburg, Germany; ^4^Department of Microbial Immune Regulation, Helmholtz Centre for Infection Research, Braunschweig, Germany; ^5^Mouse Pathology Platform, Helmholtz Centre for Infection Research, Braunschweig, Germany; ^6^Medical University Hannover, Hannover, Germany

**Keywords:** autophagy, Atg5, RORγt^+^ Foxp3^+^ Treg cells, intestinal homeostasis, inflammation

## Abstract

Autophagy is an evolutionary conserved catabolic pathway that ensures the degradation of intracellular components. The autophagic pathway is regulated by autophagy-related (Atg) proteins that govern formation of double-membraned vesicles called autophagosomes. Autophagy deficiency in regulatory T (Treg) cells leads to increased apoptosis of these cells and to the development of autoimmune disorders, predominantly characterized by intestinal inflammation. Recently, RORγt-expressing Treg cells have been identified as key regulators of gut homeostasis, preventing intestinal immunopathology. To study the role of autophagy in RORγt^+^ Foxp3^+^ Treg cells, we generated mice lacking the essential component of the core autophagy machinery Atg5 in Foxp3^+^ cells. Atg5 deficiency in Treg cells led to a predominant intestinal inflammation. While Atg5-deficient Treg cells were reduced in peripheral lymphoid organs, the intestinal RORγt^+^ Foxp3^+^ subpopulation of Treg cells was most severely affected. Our data indicated that autophagy is essential to maintain the intestinal RORγt^+^ Foxp3^+^ Treg population, thereby protecting the mice from gut inflammatory disorders.

## Introduction

Macroautophagy (hereafter called autophagy) is an ancient catabolic process that degrades bulky cargo *via* the lysosomal pathway. The cargo to be degraded may comprise protein aggregates, cell organelles or intracellular pathogens and is enclosed in double membraned vesicles, called autophagosomes (1). Autophagy is highly conserved from yeast to men and mediated by autophagy-related (ATG) proteins (1). At the core of the autophagic machinery are two ubiquitin-like conjugation systems, in which first the ubiquitin-like molecule ATG12 is conjugated to ATG5 (2). Together with ATG16L1, the ATG5-12 complex associates with nascent autophagosomes and acts as an E3 ligase to conjugate the ubiquitin-like molecule ATG8 to the lipid phosphatidylethanolamine (3). The ATG family of proteins is crucial for elongation of the nascent autophagosome and closure of the double membrane vesicle, which finally fuses with a lysosome to degrade the cargo (4).

The immune response is regulated by a specific subset of CD4^+^ T cells that is characterized by the expression of the transcription factor Foxp3 called regulatory T (Treg) cells (5, 6). Treg cells employ several immunosuppressive mechanisms including sequestration of IL-2 by high CD25 expression, secretion of inhibitory cytokines such as transforming growth factor (TGF)-β or IL-10 as well as expression of inhibitory receptors such as cytotoxic T-lymphocyte-associated protein 4 (CTLA)-4 (7). A misbalance in the ratio of Treg cells to the effector arm of the immune system might give rise to severe pathologies. For instance, too few or non-functional Treg cells result in autoimmune diseases as observed in the human immunodysregulation polyendocrinopathy enteropathy X-linked (IPEX) syndrome and the murine scurfy mutation, both characterized by non-functional Foxp3 (8–10). Treg cells can upregulate T helper (Th) lineage-specific transcription factors to regulate immune responses mediated by corresponding Th cell lineages (11). In the context of intestinal immune homeostasis, a key cell type is the retinoic acid-related orphan receptor-γt (RORγt)^+^ Foxp3^+^ Treg subpopulation (12–14). Predominantly abundant in small intestine and colon, these cells have been described as microbiota-specific Helios^-^ Foxp3^+^ Treg cells that express the Treg functional molecules CTLA-4, ICOS, CD39 and CD73 and produce the suppressive cytokine IL-10 (12–14). Moreover, their effective ability to suppress intestinal inflammation has been demonstrated (15).

Autophagy has been shown to be crucial for T cell development and for the homeostasis of naïve T cells (16). Over the last years, several studies demonstrated that autophagy plays a key role in Treg cell metabolism, function and viability (17–19). In 2016, Le Texier and colleagues reported that specific deletion of the autophagy protein Atg7 resulted in a significant reduction of Treg cell numbers causing spontaneous T cell activation in aged mice leading to enterocolitis and scleroderma (17). Moreover, they demonstrated that Treg-intrinsic autophagy promotes Treg cell survival and Treg-mediated attenuation of graft *versus* host disease after allogenic stem cell transplantation (17). In another study, the specific deletion of Atg7 or Atg5 in Foxp3^+^ cells resulted in a drastic decline of the Treg cell population and impairment of their suppressive capacity (19). Interestingly, upregulation of c-Myc, due to increased activation of mTORC1, was detected in autophagy-deficient Treg cells, leading to enhanced glycolysis (19). This metabolic dysregulation resulted in downregulation of Foxp3, Foxo3, and Bach2, essential factors for Treg cell differentiation and maintenance as well as to an aberrant production of the inflammatory cytokines IFNγ and IL-17 (19). In the same work, the authors demonstrated that autophagy is required for the ability to suppress antitumor responses, confirming the importance of autophagy for the functional capacities of Treg cells (19). A model using mice carrying a selective deletion of *Atg16l1*, another core gene of the autophagy machinery, in Foxp3^+^ cells showed loss of intestinal Treg cells and abnormally high glycolytic metabolism in the remaining Treg cells, resulting in spontaneous intestinal inflammation driven predominantly by type 2 humoral responses (18).

Despite the recent advances and accumulating evidence of a primary role for Treg cell-autophagy to control intestinal inflammation, there are no studies about the role of autophagy in RORγt^+^ Foxp3^+^ Treg cells. Our study shows the particular requirement of autophagy for the maintenance of the RORγt^+^ Foxp3^+^ Treg cells, which are essential for sustaining intestinal homeostasis.

## Materials and Methods

### Mice

*Atg5^fl/fl^* and *Foxp3^Cre^* mice were described previously (20, 21). *Atg5* ^ΔFoxp3^ and *Atg5^fl/fl^ Foxp3^Cre/wt^* mice were generated by crossing *Atg5^fl/f^* with *Foxp3^Cre^* mice. GFP-LC3 reporter mice have been described formerly (22). All mice were kept under specific pathogen-free conditions in the animal facility of the Helmholtz Centre for Infection Research, Braunschweig (23). Animal experiments and breeding were performed in accordance with the guidelines of local and national authorities.

### Cell Culture

T cells were seeded in primary T cell medium, i.e. RPMI1640 supplemented with 10% FCS (PAA Laboratories), 50 µM β-mercaptoethanol, 50 µg/ml each of penicillin and streptomycin, 1% non-essential amino acids and 1 mM sodium pyruvate (all from Life technologies). Bafilomycin A1 was obtained from Enzo Life Science. For Treg cell stimulation, 2 µg/mL anti-CD3 (145-2C11, BioLegend), 4 µg/mL anti-CD28 (37.51, Biolegend) and 10 ng/ml murine IL-2 (402-ML, R&D) were used. For *in vitro* Treg cell induction, 2 x 10^5^ FACS-sorted naive T cells (CD4^+^, CD62L^+^ and CD25^-^) were cultured in 200 µl primary T cell medium for 5 days in the presence of 1 µg/mL plate-bound anti-CD3 (145-2C11, BioLegend), 2 µg/mL anti-CD28 (37.51, Biolegend), 10µg/ml anti-IL-4 (11B11, self-purified), 10µg/ml anti-IFNγ (XMG1.2, self-purified), 10 ng/ml murine IL-2 (402-ML, R&D), and 5 ng/mL TGF-β (R&D Systems) in a flat-bottom 96-well plate.

### Histology

Liver, lung, skin and pancreas were isolated from *Atg5* ^ΔFoxp3^ and control mice. Samples were fixed in 4% formaldehyde and embedded in paraffin. Sections of 3 µm were stained with hematoxylin-eosin (HE) and evaluated by microscopy randomized and blinded to the experimental group. Criteria for the evaluation were adapted from a previous report (24). Duodenum were collected, rolled up to “swiss roles”, fixed in 4% neutrally buffered formaldehyde and embedded in paraffin according to standard histological procedures. Sections of 3 μm thickness were stained with hematoxylin-eosin (HE) and evaluated by light microscopy randomized and blinded to the experimental groups. Small intestine samples were scored for the general criteria: severity (0-3), inflammatory infiltrate (0-3), vilus atrophy (0-3), and area involved (0-3) where score 0 depicted no alteration and score 3 massive alteration in the given parameters. The scores were added up to a total of maximally 12 per section. Stomach samples fixed and stained in the same way after collected. The scoring criteria are inflammatory infiltrate (0-3) and hyperkeratosis (0-3) for non-glandular stomach, inflammatory infiltrate (0-3) and hyperplasia (0-3) for glandular stomach where score 0 depicted no alteration and score 3 massive alteration in the given parameters.

### Isolation of Lamina Propria Leukocytes (LPL)

Density gradient centrifugation using Percoll was done to isolate LPL from small intestine (SI), cecum and colon. In brief, intestinal organs were collected under specific pathogen free (SPF) condition or after *Helicobacter* species colonization. Fecal content was removed, tissues were opened longitudinally, washed with PBS and then shaken in HBSS containing 2 mM EDTA for 20 min at 37°C. Tissues were cut into small pieces and incubated with digestion solution (DMEM containing 2% fetal bovine serum (FBS), 0.4 mg/ml collagenase D, 1 U/ml dispase and 10 μg/ml DNase I) in a shaker for 30 min at 37°C. Digested tissues were filtered through a 70 µM cell strainer (Falcon) and DMEM + 5% FBS was added to inactivate enzymes. After centrifugation, cells were resuspended in 4 ml of 40% Percoll (GE Healthcare) and overlaid on 4 ml of 80% Percoll. Percoll gradient separation was performed by centrifugation at 450 g for 25 min at 20°C. Cells in the interphase were collected and used as LPL. The collected cells were then suspended in staining buffer containing PBS, 1% FBS and 2 mM EDTA. For cytokine analysis, cells were restimulated with PMA (Sigma, 10 ng/ml) and ionomycin (Sigma, 1 μg/ml) for 4 hours in DMEM medium containing 10% FCS. After 1 hour of stimulation, Brefeldin A (Sigma, 5 μg/ml) was added to block cytokine secretion.

### Flow Cytometric Analyses

For surface marker staining, cells were resuspended in 100 μl FACS buffer (2% BSA in PBS) and incubated in the presence of the respective antibodies for 15 minutes at 4°C in the dark. Afterwards, cells were washed twice with 500 μl FACS buffer and analyzed using a LSRII flow cytometer (BD Biosciences). Prior to surface staining, in the case of cell population analyses (excluding viability determinations), dead cells were excluded by incubating the cell suspension with LIVE/DEAD^®^ Fixable Blue Dead Cell Stain staining (LifeTechnologies) for 30 minutes at 4°C in the dark and subsequently washed twice with PBS; afterwards Fc receptors were blocked by 15 minutes incubation with Fc-block (CD16/32) in FACS buffer at 4˚C and washed with FACS buffer. Intracellular protein staining was performed using the Foxp3 Staining Buffer Set (Miltenyi, #130-093-142) according to the manufacturer’s instructions. Antibodies used were: CD4-PacificBlue (L3T4, Biolegend), CD8-PECy7 (53-6.7, Biolegend), CD44-APC (IM7, Biolegend), CD62L-PerCPCy5.5 (MEL-14, eBioscience), CD95-PE (Jo2, Biolegend), c-FLIP (#896537, R&D Systems), Mcl-1 (#94296, Cell Signaling Technology) and anti-GFP (FM264G, YFP-cross-reactive, Biolegend). Apoptosis was analyzed by staining with CellEvent Caspase 3/7 Green (Life Technologies). For intracellular staining of cytokines and transcription factors from lamina propria leukocytes, cells were first stained for surface markers including anti-CD45 (30-F11, Biolegend), anti-CD3 (17A2, Biolegend), anti-CD4 (RM4-5, GK1.5, Biolegend). To distinguish live dead cells AlexaFluor-350 NHS Ester (Life Technologies) was used. After that, cells were fixed in Fix/Perm buffer (eBioscience) at 4°C for 30 min, followed by permeabilization in eBioscience permeabilization buffer at 4°C for 30 min in the presence of antibodies including anti-RORγt (Q31-378, BD Biosciences), anti-GFP (FM264G, YFP-cross-reactive, Biolegend), anti-Helios (22F6, Biolegend), anti-Gata3 (TWAJ, eBioscience), anti-Foxp3 (FJK-16s, eBioscience), anti-IL-17A (TC11-18H10.1, BioLegend) and anti-IFNγ (XMG1.2, BioLegend). Samples were acquired using LSR Fortessa or LSR II (Becton, Dickinson and Company) and analyzed by FlowJo software (TreeStar Inc.).

### Proliferation Analyses by Cell Trace Violet (CTV) Staining

For proliferation analysis, MACS sorted Treg cells were stained with cell trace violet (CTV, LifeTechnologies). Briefly, Treg cells were washed two times with 1x PBS and re-suspended in 1 ml of 1x PBS. CTV was added to a final concentration of 5 µM and cells were incubated for 20 min at 37°C in the dark. Subsequently, 3 x 10^5^ Treg cells were stimulated with 2 µg/ml plate-bound anti-CD3 and 4 µg/ml soluble anti-CD28 plus 10 ng/ml IL-2. On day 3 cells were analyzed by flow cytometry.

### Western Blot Analysis

Cell lysates were obtained by incubating the samples in TPNE lysis buffer (1x PBS, 300 mM NaCl, 2 mM EDTA, 1% v/v Tritron X-100) supplemented with 1 mM phenylmethylsulfonyl fluoride, 1 µg/ml protease inhibitor mix (aprotinin, leupeptin, pepstatin A, chymostatin) and 0.4 mM sodium orthovanadate for 20 minutes on ice followed by centrifugation (15 minutes, 20.000 g). Protein concentration was determined with the BCA protein assay kit (Thermo Scientific). For electrophoresis, protein lysates were loaded onto 12% SDS-polyacrylamide gels and blotted onto a PVDF membrane (GE Healthcare). After incubation with anti-Atg5 (D1G9, Cell Signaling Technology), c-FLIP (#3210, Cell Signaling Technology), Mcl-1 (#94296, Cell Signaling Technology) or anti-β-actin (A2228, Sigma-Aldrich) proteins were detected by chemiluminescence (Thermo Scientific).

### Cytokine Determination

Cytokine levels from serum were determined using Th1/Th2 Mouse 6-Plex Panel (Life Technologies) in a Luminex^®^ instrument (Luminex Corporation) by following the manufacturer’s protocol.

### *Helicobacter* Species Colonization

WT B6 mice harboring SPF flora plus *Helicobacter* species, including *H. hepaticus*, *H. rodentium*, and *H. typhlonius* were utilized as *Helicobacter* donors. Colonization of *Atg5* ^ΔFoxp3^ and littermate control mice was conducted by cohousing or fecal transplantation. For fecal transplantation donor mice were euthanized, intestinal content was collected in BBL thioglycollate media (BD Bioscience) and homogenized by vortexing. To remove coarse particles under anaerobic conditions the content was filtered through a 70 µm sterile filter. After centrifugation (10 min, 500g, 4°C), the pellet containing fecal bacteria was resuspended in BHI medium (Sigma-Aldrich). Recipient mice were orally gavaged with a total 200 µl of fecal bacterial content. A 3-week time period was given for a successful establishment of fecal transplanted *Helicobacter* species, which was further confirmed by specific PCR of fecal bacteria and 16S rRNA gene sequencing.

### Microbial Community Analysis

Fresh stool samples of mice were collected and immediately stored at -20°C. DNA was extracted according to established protocols using a method combining mechanical disruption (bead-beating) and phenol/chloroform-based purification (25). Briefly, a sample was suspended in a solution containing 500 mL of extraction buffer (200 mM Tris, 20 mM EDTA, and 200 mM NaCl [pH 8.0]), 200 mL of 20% SDS, 500 mL of phenol: chloroform:isoamyl alcohol (24:24:1), and 100 mL of 0.1 mM zirconia/silica. Samples were homogenized twice with a bead beater (BioSpec) for 2 min. After precipitation of DNA, crude DNA extracts were resuspended in Tris-EDTA (TE) buffer with 100 µg/mL RNase and column-purified to remove PCR inhibitors (BioBasic). Amplification of the V4 region (F515/R806) of the 16S rRNA gene was performed according to previously described protocols (26). Samples were sequenced on an Illumina MiSeq platform (PE250). Filtering of sequences for low-quality reads (q > = 30) and demultiplexing were performed using QIIME v1.8.0 (27). Reads were clustered into operational taxonomical units (OTUs) based on 97% nucleotide identity of the amplicon sequences using UCLUST reference OTU picking, followed by taxonomic classification using the Ribosomal Database Project (RDP) classifier executed at 80% bootstrap confidence cut off (28, 29). Sequences without a matching reference dataset were grouped as *de novo* using UCLUST. The OTU absolute abundance table and mapping file were used for statistical analyses and data visualization in the R statistical programming environment package phyloseq (30). To determine bacterial OTUs that explained differences between microbiota settings, the LEfSe method was used (31). OTUs with Kruskal-Wallis test < 0.05 and LDA scores > 3.5 were considered informative.

### Quantification of Intestinal Lipocalin-2 (LCN2) by ELISA

LCN2 levels were estimated in the intestinal content supernatants using Duoset murine LCN2 ELISA kit (R&D Systems, USA) as described previously (32), with following modifications. Weighted intestinal lumen or tissue samples were resuspended in 1ml PBS containing 0.1% Tween 20 with 0.2ml 1mm beads. A homogenous suspension was generated by bead beating (BioSpec) for 4-5 times, 1 min each time. These samples were then centrifuged (Eppendorf, 5424R) for 5 min at 12,000 ×g and 4°C. Clear supernatants were collected and stored at −20°C until analysis. Samples or diluted samples were used in the assay according to the manufacturers’ instructions. The colorimetric peroxidase substrate tetramethylbenzidine (TMB) was quantified by measurement of absorbance at 450 and 540 nm (SpectraMax M3, Molecular Devices). Standards and samples were fitted using a non-linear regression and lipocalin-2 values were calculated back to ng of lipocalin-2 per g of sample.

### Statistical Analysis

Statistical significance was calculated by Mann-Whitney or Kruskal–Wallis one-way ANOVA tests using Prism Software (GraphPad Software, La Jolla, CA). P values considered significant as follows: *p < 0.05; **p < 0.01; ***p < 0.001 and ****p < 0.0001.

## Results

### Regulatory T Cells Display Active Autophagy

**Figure 1:**
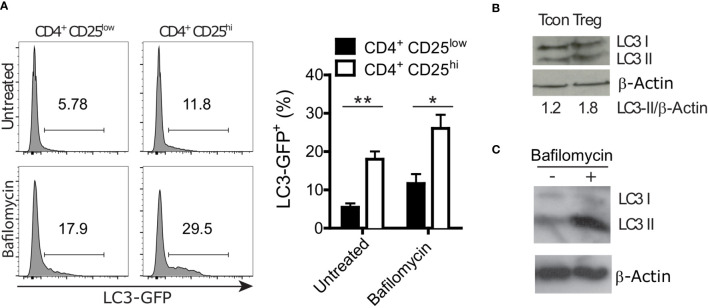
Regulatory T (Treg) cells show a higher autophagy rate than conventional CD4^+^ T (Tcon) cells. **(A)** Representative histograms (left) and statistical summary (right) of flow cytometric GFP fluorescence analysis of Bafilomycin-treated (2 hours) and untreated CD4^+^ CD25^low^ and CD4^+^ CD25^high^ lymph node cells (n=5 each) from GFP-LC3 mice. Bar graphs represent the mean ± SEM. Statistical analyses were performed by two-tailed Mann-Whitney tests. **(B)** Immunoblot analysis of LC3 in FACS-purified Tcon (CD4^+^ CD25^low^) and Treg (CD4^+^ CD25^high^) cells. Numbers show normalized quantification of LC3 II relative to β-Actin band intensity. Quantification was performed using ImageJ 1.44p software. **(C)** Immunoblot analysis of LC3 in *in vitro* differentiated Treg cells treated with 100 nM Bafilomycin for 2 hours or left untreated. *p < 0.05, **p < 0.01.

Autophagy is involved in both selective degradation of cytoplasmic organelles and in bulk degradation of primarily long-lived cytoplasmic proteins. Autophagic degradation activity is often measured by the autophagic flux. Experimentally, the flux is quantified by blocking the degradation of autophagic cargo in the lysosome and measuring the rate of autophagosome accumulation compared to the not inhibited condition (33). We took advantage of GFP-LC3 reporter mice to measure LC3 accumulation in conventional T cells (CD4^+^ CD25^low^) and Treg (CD4^+^ CD25^hi^) cells by flow cytometry. Cells were treated for 2 hours with Bafilomycin A1, an inhibitor of the autophagic flux that is preventing the acidification of lysosomes and endosomes (33), or left untreated. In both conditions, we found higher LC3 accumulation in Treg cells than in conventional T cells (**Figure 1A**). Furthermore, we analyzed endogenous LC3-II, which is formed by conjugation of LC3 to phosphatidylethanolamine and a widely used marker of autophagosomal membranes (34). In line with the GFP-LC3 data, immunoblot analyses revealed higher LC3-II levels in sorted Treg cells compared to conventional CD4^+^ cells (**Figure 1B**). Moreover, Bafilomycin A1-treated *in vitro* differentiated Treg cells displayed more LC3-II than untreated Treg cells (**Figure 1C**). In line with other reports (17–19), these data indicate that Treg cells have a prominent autophagic activity.

### Signs of Incipient Immune Homeostasis Disruption in the Gut of Young *Atg5^ΔFoxp3^* Mice

**Figure 2:**
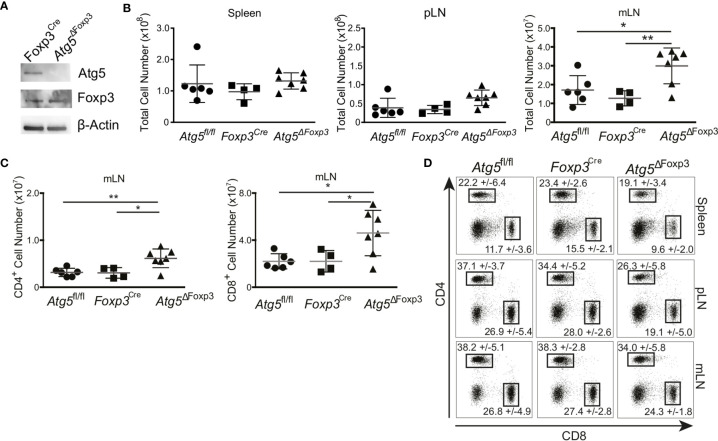
Incipient intestinal immune system alteration of young *Atg5* ^ΔFoxp3^ mice. **(A)** Immunoblot analysis of Atg5 in FACS-purified Treg cells from *Foxp3^Cre^* and *Atg5* ^ΔFoxp3^ mice. **(B)** Total cell numbers of spleen, peripheral lymph nodes (pLN) and mesenteric lymph nodes (mLN) from 8-week-old *Atg5^fl/f^, Foxp3^Cre^* and *Atg5* ^ΔFoxp3^ mice. **(C)** Total cell numbers of CD4^+^ and CD8^+^ T cell populations from mesenteric lymph nodes (mLN) of 8 weeks old *Atg5* ^fl/fl^, *Foxp3^Cre^* and *Atg5* ^ΔFoxp3^ mice. **(D)** Representative dot plots of CD4- and CD8-expressing cell populations in 8-weeks-old *Atg5^fl/fl^*, *Foxp3^Cre^* and *Atg5* ^ΔFoxp3^ mice. Mean and SD are indicated; n=4-7. **(B, C)** The Kruskal–Wallis one-way ANOVA tests were used for statistical analysis. Mean ± SEM are given. Each symbol represents a single mouse in scatter plots. *p < 0.05, **p < 0.01.

Atg5 participates in the formation of the autophagosome and is an essential component of the canonical autophagy machinery (35). To test the significance of autophagy in Treg cells, we crossed mice carrying loxP-flanked Atg5 with mice expressing the recombinase protein Cre under the control of the Foxp3 promoter (**Supplementary Figure 1A**). As expected, Atg5 was absent in the Treg cells of the conditional knockout mice (hereafter referred to as *Atg5^ΔFoxp3^*) but not in *Foxp3Cre* control mice (**Figure 2A**). We next characterized the phenotype of young (8-week-old) *Atg5^ΔFoxp3^* mice. While we did not observe any macroscopic alteration and significant cellularity variation in the spleen and peripheral lymph nodes (pLN), we detected enlarged mesenteric lymph nodes (mLN) and increased cellularity in mLN of young *Atg5^ΔFoxp3^* mice (**Figure 2B**). Accordingly, the number of CD4^+^ and CD8^+^ T cells in the mLN of young *Atg5^ΔFoxp3^* mice was greater than in the control animals (**Figure 2C**). Frequencies of CD4^+^ and CD8^+^ T cells were rather lower in spleen and pLN and similar in mLN in young *Atg5^ΔFoxp3^* mice compared to the controls (**Figure 2D**), indicating that the high numbers of T cells in mLN are not caused by the inflation of those particular cell types but due to organ thickening. Mice at this age showed no obvious behavioral change.

### Selective Deletion of *Atg5* in Treg Cells Results in Systemic Inflammation

**Figure 3:**
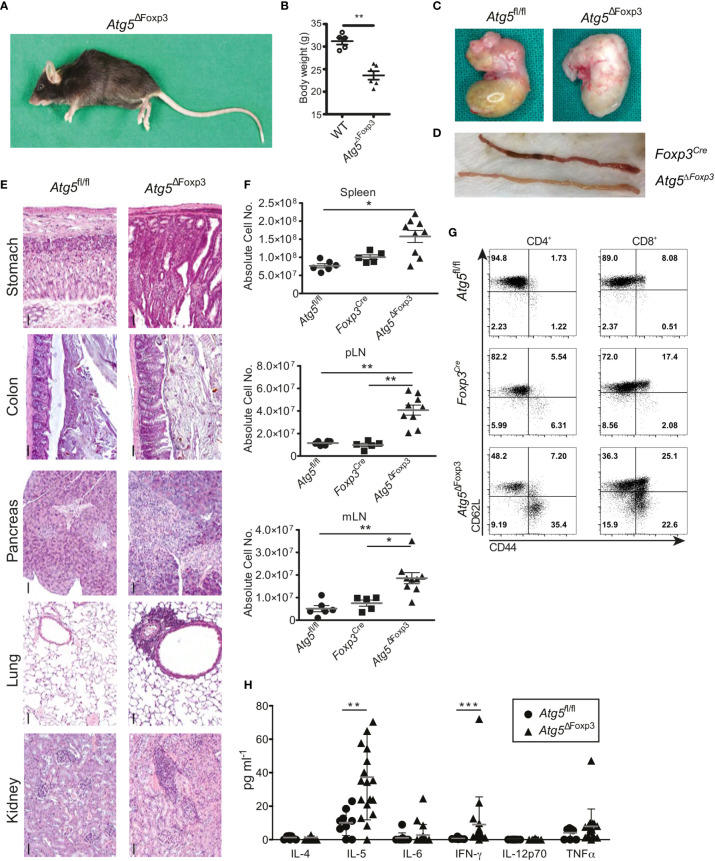
Mature *Atg5* ^ΔFoxp3^ mice develop systemic inflammation. **(A)** Picture of a 22-week-old *Atg5* ^ΔFoxp3^ mouse. **(B)** Body weight of *Atg5* ^ΔFoxp3^ and BL6 (control) mice at the age of 22 weeks. **(C)** Stomach pictures of *Atg5* ^fl/fl^ (left) and *Atg5* ^ΔFoxp3^ (right) mice. **(D)** Picture of the small intestine from *Foxp3^Cre^* and *Atg5* ^ΔFoxp3^ mice. **(E)** Representative histological sections of paraffin-embedded tissue from kidney, lung, pancreas, colon and stomach of 22-week-old *Atg5^fl/fl^* and *Atg5* ^ΔFoxp3^ mice stained with hematoxylin and eosin. Bar size: 50 µm **(F)** Absolute cell numbers of spleen, peripheral lymph nodes (pLN) and mesenteric lymph nodes (mLN) from 11-20-week-old *Atg5^fl/fl^*, *Foxp3^Cre^* and *Atg5* ^ΔFoxp3^ mice. Mean ± SEM are given. The Kruskal–Wallis one-way ANOVA tests were used for statistical analysis. Each symbol represents a single mouse in scatter plots. **(G)** Representative dot plots of T cell activation markers CD44 and CD62L in CD4^+^ Foxp3^-^ and CD8^+^ cells from peripheral lymph nodes of 11-20-week-old *Foxp3^Cre^* and *Atg5* ^ΔFoxp3^ mice (n=4 Foxp3^Cre^ and n=8 *Atg5* ^ΔFoxp3^ mice). **(H)** Cytokine concentrations in sera from 11-20-week-old *Atg5^fl/fl^* and *Atg5* ^ΔFoxp3^ mice measured by Luminex^®^ technology. **(B, H)** Statistical analyses were performed by two-tailed Mann-Whitney tests. *p < 0.05, **p < 0.01, ***p < 0.001.

As the *Atg5^ΔFoxp3^* mice got older, they adopted a lethargic attitude, a hunched posture (**Figure 3A**) and delayed growth evidenced by low body weight (**Figure 3B**). Moreover, the stomach appeared swollen and pale in *Atg5^ΔFoxp3^* mice reminiscent of autoimmune gastritis (**Figure 3C**). Similarly, the small intestine was pale (**Figure 3D**) showing a diffuse lymphocytic infiltrate and vilus atrophy in histology (data not shown****). Perivascular lymphocytic infiltration consistent with systemic autoimmunity was also found in all organs analysed including colon, pancreas, lung and kidney (**Figure 3E**) accompanied by increased cellularity and enlargement of peripheral lymphoid organs (**Figure 3F** and **Supplementary Figure 1B**). Accordingly, CD4^+^ and CD8^+^ T cells of *Atg5^ΔFoxp3^* mice showed a highly activated phenotype, based on the expression pattern of the CD62L and CD44 markers (**Figure 3G**). These two T cell lineages presented reduced frequencies in lymph nodes, probably because of the rise of other effector immune cell populations (**Supplementary Figure 1C**). Furthermore, elevated levels of pro-inflammatory cytokines, particularly significant IL-5 and IFN-γ, were found in the blood of these animals (**Figure 3H**). Taken together, these data indicate that deficiency of Atg5 in Treg cells promotes hyper-activation of the immune system with a prominent impact on the gastrointestinal tract.

### Reduced Viability of Atg5-Deficient Treg Cells

**Figure 4:**
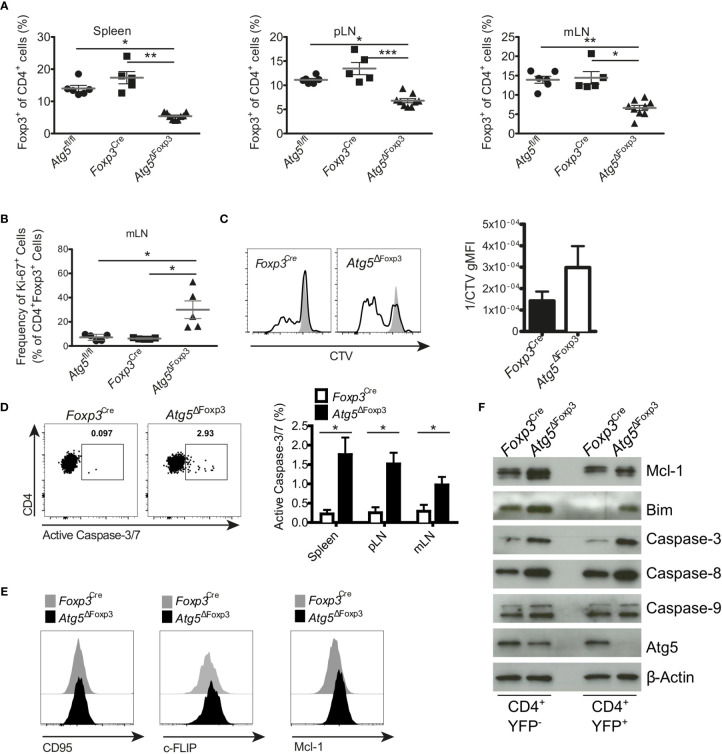
Enhanced apoptosis in Atg5-deficient Treg cells. **(A)** Percentages of CD4^+^ Foxp3^+^ cells of spleen, peripheral lymph node (pLN) and mesenteric lymph node (mLN) from 11-20-week-old *Atg5^fl/fl^, Foxp3^Cre^* and *Atg5* ^ΔFoxp3^ mice. **(B)** Frequency of Ki-67^+^ within CD4^+^ Foxp3^+^ cells from mesenteric lymph nodes of *Atg5^fl/fl^, Foxp3^Cre^* and *Atg5* ^ΔFoxp3^ mice. **(C)** Cell trace violet (CVT) labeled Treg cells from *Atg5* ^ΔFoxp3^ and *Foxp3^Cre^* mice were stimulated *in vitro* with anti-CD3, anti-CD28 and IL-2 (black line), or left unstimulated (grey). Proliferation as indicated by CVT dilution was quantified on day 3 *via* flow cytometry. Bar graph shows Mean ± SEM of 2 independent experiments. **(D)** Representative dot plots and summary bar graph of active Caspase-3/7 staining in Treg cells of *Foxp3^Cre^* and *Atg5* ^ΔFoxp3^ mice. Bar graph shows mean ± SEM (n=4 each); Statistical analyses were performed by two-tailed Mann-Whitney tests. **(E)** Histograms of CD95, c-FLIP and Mcl-1 expression in Treg cells from *Atg5* ^ΔFoxp3^ (black) and *Foxp3^Cre^* mice (grey) mice determined by flow cytometry. **(F)** Immunoblot analysis of apoptosis-related proteins in FACS-purified Tcon (CD4^+^ YFP^-^) and Treg (CD4^+^ YFP^+^) cells from *Atg5* ^ΔFoxp3^ and *Foxp3^Cre^* mice. **(A, B)** The Kruskal–Wallis one-way ANOVA tests were used for statistical analysis. Mean ± SEM are given. Each symbol represents a single mouse in the scatter plots. *p < 0.05, **p < 0.01, ***p < 0.001.

Since *Atg5^ΔFoxp3^* mice developed an inflammatory phenotype and Treg cells are essential to keep immune homeostasis (36), we were prompted to investigate the Treg cells in these mice in more detail. Flow cytometric analyses showed that the *Atg5^ΔFoxp3^* mice displayed reduced Treg cell frequencies in peripheral lymphoid organs compared to the controls (**Figure 4A** and **Supplementary Figure 2A**). However, no obvious differences in the absolute numbers of Treg cells were detected because of the organ enlargement and the increased absolute cellularity in *Atg5^ΔFoxp3^* mice (**Supplementary Figure 2B**). To determine the cause for reduced Treg cell frequencies in *Atg5^ΔFoxp3^* mice, we measured expression of the Ki-67 proliferation marker in Treg cells. Intracellular flow cytometric analyses showed that *Atg5^ΔFoxp3^* mice contained high amounts of Ki-67-expressing Treg cells whereas they remained low in all controls (**Figure 4B**). We next tested the proliferative capacity of isolated Treg cells *in vitro* from *Atg5^ΔFoxp3^* mice and *Foxp3Cre* control mice by fluorescent cell tracking dye dilution. In line with our previous findings, Atg5-deficient Treg cells displayed higher proliferation upon CD3/28 + IL-2 *in vitro* stimulation (**Figure 4C**). These data suggest that the reduced Treg cell population in *Atg5^ΔFoxp3^* mice is not due to a cell proliferation defect. Since apoptosis decisively maintains Treg cell homeostasis (36), we inspected cell death of the Treg cells. Remarkably, Atg5-deficient Treg cells from peripheral lymphoid organs showed a higher apoptosis rate than Treg cells from control mice as indicated by active caspase-3 detected in these cells *via* flow cytometry (**Figure 4D**). Apoptosis is a highly regulated process in Treg cells (36). In particular, IL-2-driven Mcl-1, an anti-apoptotic protein related to the mitochondrial pathway, is essential for maintaining Treg survival (37). Furthermore, these cells are susceptible to CD95-mediated apoptosis and anti-apoptotic c-FLIP proteins are necessary to prevent loss of Treg cells (38). Thus, we analyzed the expression levels of Mcl-1, c-FLIP and CD95 by flow cytometry. The expression of the death receptor CD95 and c-FLIP was equivalent in Atg5-deficient Treg cells and Atg5-proficient Treg cells (**Figure 4E**). Surprisingly, Treg cells from *Atg5^ΔFoxp3^* mice showed higher expression of the anti-apoptotic protein Mcl-1 than control mice (**Figure 4E**), suggesting that loss of autophagy in Treg cells does not lead to sensitization of the intrinsic apoptosis pathway. Next, to further dissect the contribution of the apoptosis pathways to the impaired Treg viability, apoptosis-related protein expression was determined in sorted CD4^+^ Tcon and Treg cells from *Atg5^ΔFoxp3^* and *Foxp3Cre* control mice by immunoblotting. In line with the flow cytometry data, Mcl-1 was higher expressed in Treg cells from the *Atg5^ΔFoxp3^* compared to *Foxp3Cre* mice (**Figure 4F**). Consistent with previous reports (19), the pro-apoptotic protein Bim was also significantly higher in the knockout cells, suggesting an explanation for the higher apoptosis rate in Treg cells of *Atg5^ΔFoxp3^* mice, despite their high levels of the anti-apoptotic protein Mcl-1. In agreement with the flow cytometric data, the levels of the apoptotic effector Caspase-3 were elevated in Atg5-deficient Treg cells (**Figure 4F**). Surprisingly, Caspase-9 expression was identical in Treg cells from *Atg5^ΔFoxp3^* mice compared to Treg cells from the controls, whereas Caspase-8 was significantly elevated in the deficient cells (**Figure 4F**). These findings suggest a role of the death receptor-mediated apoptosis pathway in the elimination of Treg cells in *Atg5^ΔFoxp3^* mice.

### Impaired Foxp3^+^ RORγt^+^ Treg Cell Presence in *Atg5^ΔFoxp3^* Mice

**Figure 5:**
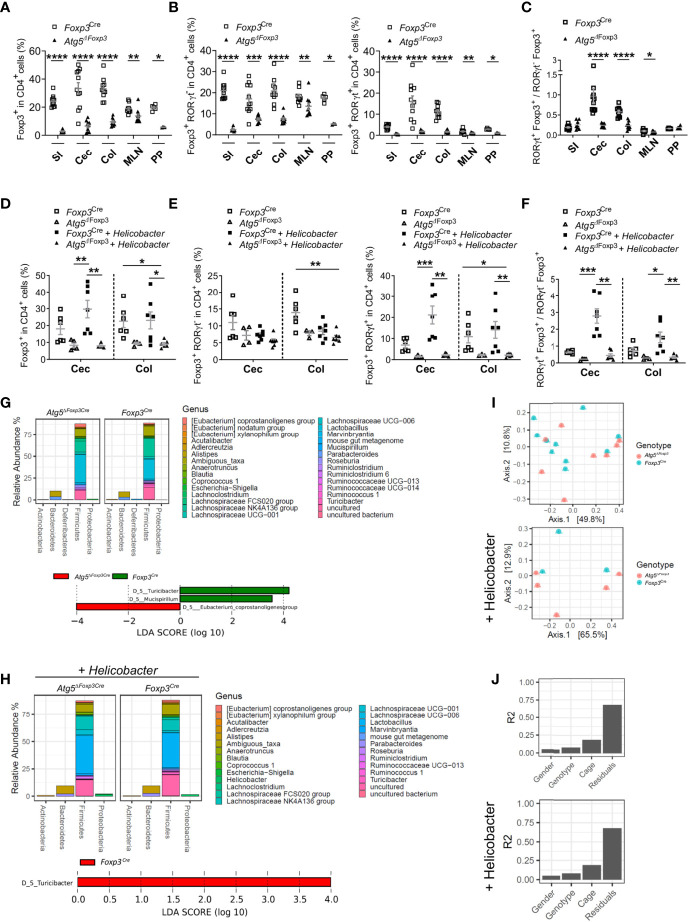
Atg5 deficiency affects dramatically intestinal Foxp3^+^ RORγt^+^ Treg cells. **(A)** Frequencies of Foxp3^+^ cells within the CD4^+^ cell populations in small intestine (SI), cecum (Cec), colon (Col), mesenteric lymph nodes (MLN) and Peyer´s patches (PP) of *Foxp3^Cre^* and *Atg5* ^ΔFoxp3^ mice. **(B)** Frequencies of Foxp3^+^ RORγt^-^ cells (left) and Foxp3^+^ RORγt^+^ (right) within the CD4^+^ cell populations in small intestine (SI), cecum (Cec), colon (Col), mesenteric lymph nodes (MLN) and Peyer´s patches (PP) of *Foxp3^Cre^* and *Atg5* ^ΔFoxp3^ mice. **(C)** Ratio of Foxp3^+^ RORγt^+^ and Foxp3^+^ RORγt^-^ cells in small intestine (SI), cecum (Cec), colon (Col), mesenteric lymph nodes (MLN) and Peyer´s patches (PP) of *Foxp3^Cre^* and *Atg5^ΔFoxp3^* mice. Mean ± SEM are given. **(D)** Frequencies of Foxp3^+^ cells within CD4^+^ cells in cecum (Cec) and colon (Col) of *Foxp3^Cre^* and *Atg5* ^ΔFoxp3^ mice under specific-pathogen-free conditions and colonized with *Helicobacter* species. **(E)** Frequencies of Foxp3^+^ RORγt^-^ cells (left) and Foxp3^+^ RORγt^+^ (right) cells within the CD4^+^ cell populations in cecum (Cec) and colon (Col) of *Foxp3^Cre^* and *Atg5* ^ΔFoxp3^ mice under specific-pathogen-free conditions and colonized with *Helicobacter* species. **(F)** Ratio Foxp3^+^ RORγt^+^ *vs*. Foxp3^+^ RORγt^-^ cells in cecum (Cec) and colon (Col) of *Foxp3^Cre^* and *Atg5* ^ΔFoxp3^ mice under specific-pathogen-free conditions and colonized with *Helicobacter* species. **(G)** Representation of relative abundances of bacterial genus (top) and analysis of differentially abundant bacterial genus by LEfSe (linear discriminant analysis effect size) (bottom) present in fecal microbiota of *Foxp3^Cre^* and *Atg5* ^ΔFoxp3^ mice under specific-pathogen-free conditions using 16S rRNA sequencing **(H)** Representation of relative abundances of bacterial genus (top) and analysis of differentially abundant bacterial genus by LEfSe (linear discriminant analysis effect size) (bottom) present in fecal microbiota of *Foxp3^Cre^* and *Atg5* ^ΔFoxp3^ mice colonized with *Helicobacter* using 16S rRNA sequencing. **(I)** Analysis of β-diversity (principal coordinates analysis (PCoA)) and **(J)** permutational multivariate analysis of variance (ADONIS) of fecal microbiota from *Foxp3^Cre^* and *Atg5* ^ΔFoxp3^ mice under specific-pathogen-free conditions (top) and colonized with *Helicobacter* (bottom) using 16S rRNA sequencing. **(A–C)** Statistical analyses were performed by two-tailed Mann-Whitney tests. **(D–F)** The Kruskal–Wallis one-way ANOVA tests were used for statistical analysis. **(A–F)** Mean ± SEM are given. Each symbol represents a single mouse in the scatter plots. *p < 0.05, **p < 0.01, ***p < 0.001, ****p < 0.0001.

Because Atg5-deficiency in Treg cells caused massive inflammation in the gastrointestinal tract and Treg cells expressing RORγt are essential to control intestinal inflammation (15), we investigated whether this Treg cell subpopulation was altered in *Atg5^ΔFoxp3^* mice. In line with the systemic Treg cell reduction, *Atg5^ΔFoxp3^* mice displayed a notably low level of Treg cells in the intestines, mLN and Peyer´s patches (**Figure 5A**). When we analysed intestinal Treg cell subpopulations based on RORγt expression, we observed that the Foxp3^+^ RORγt^+^ Treg cells were barely detectable within the aforementioned organs of *Atg5^ΔFoxp3^* mice (**Figure 5B**). In contrast, despite their reduced presence, we found Foxp3^+^ RORγt^-^ Treg cells in the intestines and the associated lymphoid tissues examined (**Figure 5B**). For example, the Foxp3^+^ RORγt^+^ Treg cell population displayed a 6.8-fold reduction in the colon of *Atg5^ΔFoxp3^* mice compared to the control mice and, in contrast, the Foxp3^+^ RORγt^-^ Treg cell population presented a 2.8-fold reduction (**Figure 5B**). In summary, there was a noteworthy lack of Foxp3^+^ RORγt^+^ Treg cells within the composition of the intestinal Treg cell compartment, being particularly unbalanced in the colon and cecum of *Atg5^ΔFoxp3^* mice (**Figure 5C**).

Since Foxp3^+^ RORγt^+^ Treg cells are microbiota-specific Treg cells that can be induced in response to pathobionts (39), we performed intestinal colonization with a mix of *Helicobacter* species in *Atg5^ΔFoxp3^* mice. While *Helicobacter* colonization increased Treg frequencies in the intestine of control animals, the frequencies of these cells remained remarkably low in *Helicobacter*-colonized and non-colonized *Atg5^ΔFoxp3^* mice (**Figure 5D**). Dissecting the Treg cell compartment, the Foxp3^+^ RORγt^+^ Treg subpopulation of *Atg5^ΔFoxp3^* mice was unresponsive to the *Helicobacter* colonization, remaining at virtually undetectable levels (**Figure 5E**). Interestingly, we observed that *Helicobacter* colonization had practically no impact on the levels of the Foxp3^+^ RORγt^-^ Treg population (**Figure 5E**). Focusing on the colon of the *Helicobacter*-colonized groups, the *Atg5^ΔFoxp3^* mice had a 6.7-fold reduction in the Foxp3^+^ RORγt^+^ Treg cell population compared to the control group, whereas the Foxp3^+^ RORγt^-^ Treg cells showed only a 1.3-fold reduction compared to the control mice (**Figure 5E**). The ratio of Foxp3^+^ RORγt^+^ *vs*. Foxp3^+^ RORγt^-^ Treg cells evidenced that autophagy competent Foxp3^+^ RORγt^+^ Treg cells can be induced in the gut, whereas the *Atg5^ΔFoxp3^* mice have a reduced capacity to adequately induce or maintain intestinal Foxp3^+^ RORγt^+^ Treg cells (**Figure 5F**). This inability may explain the gastrointestinal tract-specific tissue injury found in *Helicobacter*-colonized as well as non-colonized *Atg5^ΔFoxp3^* mice (**Supplementary Figure 3A**) and their predisposition to develop spontaneous gut inflammation. IL-5-mediated eosinophil activation has been related to colitis development (40), which may explain the elevated levels of IL-5 in the blood of *Atg5^ΔFoxp3^* mice. Classically, dysregulated Th1 and Th17 cell as well as innate lymphoid cell responses characterizes chronic intestinal inflammation (41). Further analysis showed no raise in IFNγ-producing T cell frequencies, but elevated frequencies of IL-17A-producing T cells and Foxp3^-^ RORγt^+^ cells in the gut and associated lymphoid tissues of *Atg5^ΔFoxp3^* mice (**Supplementary Figures 3B, C**). Besides the conceivable IL-5-mediated immune response, these data indicate an important contribution of Th17-mediated immune responses to the intestinal inflammation of *Atg5^ΔFoxp3^* mice, consistent with the ability of intestinal Foxp3^+^ RORγt^+^ Treg cells to antagonize Th17 inflammatory responses (12, 14).

Changes in the intestinal mucosal flora have been associated with inflammatory bowel disease (42). Therefore, we wanted to investigate whether the gut inflammation afflicting the *Atg5^ΔFoxp3^* mice was reflected in their microbiome. Besides the reduction of the Turicibacter, which belongs to the Erysipelotrichaceae bacterial family, the microbiota composition remained reasonably stable in *Atg5^ΔFoxp3^* mice compared to the *Foxp3^Cre^* control mice under specific-pathogen-free conditions as well as in mice colonized with *Helicobacter* species (**Figures 5G, H** and **Supplementary Figure 3D**). Alpha diversity and Shannon index analyses also reflected a minor reduction in the microbiota diversity of *Atg5^ΔFoxp3^* mice (**Supplementary Figure 3E**). Principal component analysis (PCA) showed that the individual samples of the two groups were intermixed (**Figure 5I**). These data, together with the results of the permutational multivariate analysis of variance (ADONIS) (**Figure 5J**), indicate that differences in the microbiota are not dependent on the mouse genotype.

### *Atg5* Deletion Intrinsically Affects the Intestinal RORγt^+^ Foxp3^+^ Cell Population

**Figure 6:**
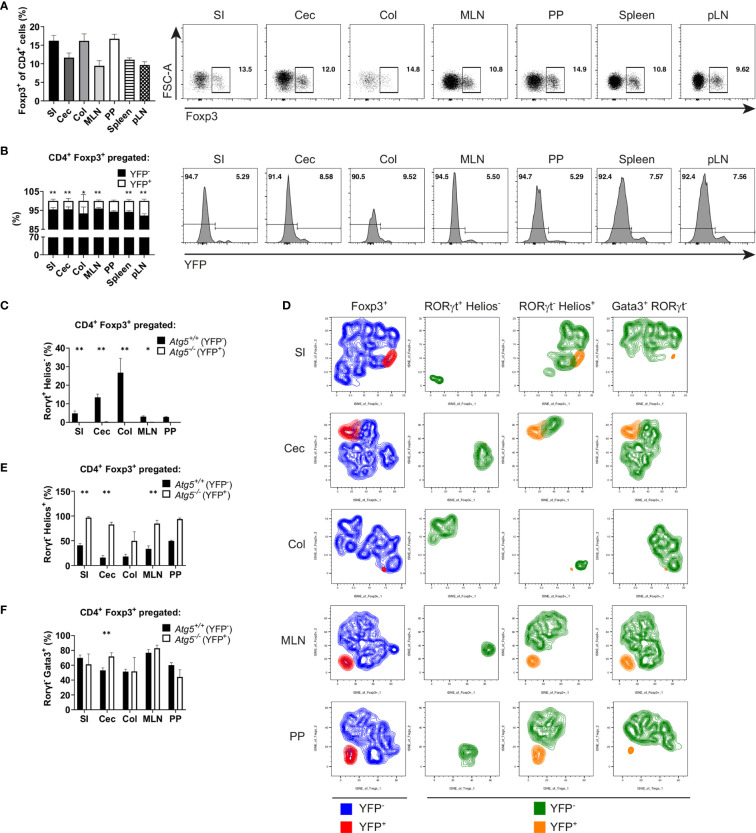
Atg5 is crucial to preserve the intestinal Foxp3^+^ RORγt^+^ Treg cell population. **(A)** Bar graph (left) and representative dot plots (right) of Foxp3^+^ cell frequencies within CD4^+^ cells of small intestine (SI), cecum (Cec), colon (Col), mesenteric lymph nodes (MLN), Peyer´s patches (PP), spleen and peripheral lymph nodes (pLN) of *Atg5^fl/fl^ Foxp3^Cre/wt^* mice. **(B)** Bar graph (left) and representative dot plots (right) of YFP^-^ and YFP^+^ distribution within the CD4^+^ Foxp3^+^ cells of small intestine (SI), cecum (Cec), colon (Col), mesenteric lymph nodes (MLN), Peyer´s patches (PP), spleen and peripheral lymph nodes (pLN) of *Atg5^fl/fl^ Foxp3^Cre/wt^* mice. **(C)** Atg5-proficient (YFP^-^) and Atg5-deficient (YFP^+^) frequencies within the RORγt^+^ Helios^-^ Treg cells in small intestine (SI), cecum (Cec), colon (Col), mesenteric lymph nodes (MLN) and Peyer´s patches (PP) of *Atg5^fl/fl^ Foxp3^Cre/wt^* mice. **(D)** tSNE plots of YFP^-^ and YFP^+^ cell distribution within the Foxp3^+^, RORγt^+^ Helios^-^, RORγt^-^ Helios^+^ and RORγt^-^ Gata3^+^ cells in small intestine (SI), cecum (Cec), colon (Col), mesenteric lymph nodes (MLN) and Peyer´s patches (PP) of *Atg5^fl/fl^ Foxp3^Cre/wt^* mice. **(E)** Atg5-proficient (YFP^-^) and Atg5-deficient (YFP^+^) frequencies within the RORγt^-^ Helios^+^ Treg cells in small intestine (SI), cecum (Cec), colon (Col), mesenteric lymph nodes (MLN) and Peyer´s patches (PP) of *Atg5^fl/fl^ Foxp3^Cre/wt^* mice. **(F)** Atg5-proficient (YFP^-^) and Atg5-deficient (YFP^+^) frequencies within the RORγt^-^ Gata3^+^ Treg cells in small intestine (SI), cecum (Cec), colon (Col), mesenteric lymph nodes (MLN) and Peyer´s patches (PP) of *Atg5^fl/fl^ Foxp3^Cre/wt^* mice. **(B, C, E, F)** Statistical analyses were performed by two-tailed Mann-Whitney tests. Mean ± SEM are given. (SI, n=6; Cec, n=6; Col, n=4, MLN, n=5; PP, n=3; Spleen, n=6; pLN, n=6; data obtained from two independent experiments). *p < 0.05, **p < 0.01.

*Atg5^ΔFoxp3^* mice developed systemic inflammation, particularly focused in the intestinal tract. In order to analyze whether the reduction in the Treg cell population of *Atg5^ΔFoxp3^* mice is due to the inflammatory environment or due to an intrinsic effect, we took advantage of 14-week-old *Atg5^fl/fl^ Foxp3^Cre/wt^* female mice. Because *Foxp3* is encoded on the X chromosome and random inactivation of the X chromosome occurs in female mice, these animals contain two Treg cell populations: one expressing the Cre-YFP protein, and thus, presenting *Atg5* gene deletion, and another population expressing only the *Foxp3* wild type allele and carrying an intact *Atg5* gene. In this scenario, the *Atg5^fl/fl^ Foxp3^Cre/wt^* mice showed normal Treg cell frequencies (**Figure 6A**) and no signs of inflammation (**Supplementary Figures 4A–C**). Interestingly, inspecting the ratio of Atg5-proficient (YFP^-^) *vs*. Atg5-deficient (YFP^+^) cells within the Treg cell population, the YFP^+^ cell population was remarkably underrepresented (**Figure 6B**) indicating a notable disadvantage for the autophagy-deficient cells within the Treg cell compartment. Since our previous findings indicated a prominent deficiency of intestinal RORγt^+^ Foxp3^+^ cells in *Atg5^ΔFoxp3^* mice, we investigated the distribution of Treg cell subpopulations in the intestines of *Atg5^fl/fl^ Foxp3^Cre/wt^* female mice. In line with our previous data, the RORγt^+^ Helios^-^ Treg cell subpopulation was virtually absent within the Atg5-deficient cell fraction of the examined organs, while it was present within the Atg5-proficient Treg cell fraction (**Figures 6C, D**). In contrast, the thymic-derived RORγt^-^ Helios^+^ Treg cell subpopulation was detectable in both cell fractions (**Figure 6E**), despite its underrepresentation in the Atg5-deficient portion (**Figure 6D**). In addition, we examined the distribution of a fraction of Treg cells, which co-expresses the transcription factor Gata3 and has been related to tissue repair processes (43). Despite their reduction in the total Foxp3^+^ compartment, these cells were present within the Atg5-deficient Treg cell population in *Atg5^fl/fl^ Foxp3^Cre/wt^* mice (**Figures 6D, F**). Altogether, our results demonstrate that the deficiency of the autophagy core protein Atg5 is particularly important for RORγt^+^ Foxp3^+^ cells.

## Discussion

The intestinal mucosa is constantly exposed to commensal bacteria, pathogens as well as food-derived antigens. In order to preserve the intestinal homeostasis, inflammatory responses to dietary antigens and commensal flora must be avoided. At the same time, pathogen growth and invasion must be restricted. Infectious agents can be sensed by dendritic and intestinal epithelial cells, leading to immune effector responses (41). Hence, a delicate balance between pro- and anti-inflammatory mechanisms is essential to avoid reactions towards harmless signals and remain responsive to pathogens. Perturbations to this equilibrium may lead to the rise of food allergies or the development of inflammatory bowel disease (41, 44). RORγt^+^ Foxp3^+^ Treg cells contribute significantly to the establishment of the intestinal immune tolerance, preventing adverse inflammatory responses at intestinal mucosal interfaces (12–15). Upon immunization, RORγt^+^ Foxp3^+^ Treg cells, with a thymic origin, can be found in lymph nodes (45). However, intestinal RORγt^+^ Foxp3^+^ Treg cells arise instead by means of peripheral Treg (pTreg) cell development driven by microbiota-derived antigens (13, 14). Hence, it is reasonable to think that intestinal RORγt^+^ Foxp3^+^ Treg cells correspond to the formerly described colonic Foxp3^+^ Treg cells, which express microbiota-reactive T cell receptors and develop extra-thymically (46).

Several studies demonstrated that Treg-selective autophagy deficiency leads to a reduction in the Treg cell population and the development of spontaneous autoimmune disorders (17–19). In agreement with our findings, the intestinal tract is affected in mice lacking *Atg7* or *Atg16l1* specifically in Treg cells (17, 18), pointing out the importance of autophagy for Treg cells that maintain intestinal homeostasis. Bearing in mind these observations, we investigated the role of autophagy in intestinal Treg cells. In contrast to the reduced, but detectable peripheral Foxp3^+^ Treg cell population, RORγt^+^ Foxp3^+^ Treg cells were barely detectable in *Atg5* ^ΔFoxp3^ mice. Importantly, this was also the case in *Atg5^fl/fl^ Foxp3^Cre/wt^* mice, which due to random X chromosome inactivation should contain equal amounts of *Atg5*-proficient and -deficient Treg cells. However, more than 90% of Treg cells in these mice were of the *Atg5*-proficient genotype. Therefore, in this competitive environment, *Atg5*-deficient Treg cells have a clear disadvantage. We conclude that autophagy is required in a cell intrinsic manner for either differentiation or maintenance of RORγt^+^ Foxp3^+^ Treg cells. Furthermore, *Atg5* ^ΔFoxp3^ mice failed to produce intestinal RORγt^+^ Foxp3^+^ Treg cells upon *Helicobacter* colonization. Absence or total functional deficiency of Treg cells leads to a fulminant autoimmune disease, typically exemplary as the *scurfy* mice (9). In line with other reports (17–19), we observed that mice harboring autophagy-deficient Treg cells did not exhibit autoimmunity symptoms within the first weeks of life but rather they developed inflammatory disorders at a more mature age. Thymus-derived Treg cells restrain auto-reactive cells preventing systemic and tissue-specific autoimmunity, whereas extra-thymic generated Treg cells control inflammation at mucosal interfaces (47). That fact and the virtual absence of intestinal RORγt^+^ Foxp3^+^ Treg cells in *Atg5* ^ΔFoxp3^ mice may explain the predisposition of mice harboring autophagy-deficient Treg cells to develop spontaneous intestinal inflammation. Altogether, these findings indicate that autophagy plays a prominent role in intestinal RORγt^+^ Foxp3^+^ Treg cells.

We and others (17, 19) detected increased proliferation of autophagy-deficient Treg cells. However, this enhanced proliferation did not overcome the reduced Treg cell frequencies. Treg cell homeostasis is tightly controlled by the IL-2-regulated Mcl-1-Bim axis (37). Expression analysis of key regulators of apoptosis on the transcriptional and protein level displayed no differential expression of Bim, Bax and other Bcl-2 genes in *Atg16l1*-deficient Treg cells (18). In contrast, we and Wei et al. detected an increase of the pro-apoptotic protein Bim in autophagy-deficient Treg cells (19). Furthermore, Le Texier (17) and our data showed an increase of Mcl-1 in the same cells, which may counteract Bim activity. Despite these discrepancies, there is overwhelming evidence that autophagy deficiency results in enhanced apoptosis in Treg cells contributing to the reduction in Treg cell numbers. Interestingly, we detected impaired immune homeostasis in mesenteric lymph nodes already in young mice, while other organs were affected in older mice. This suggests that the initial gut inflammation, presumably caused by the absence of Foxp3^+^ RORγt^+^ Treg cells, instigates a systemic Treg cell reduction, enhancing the systemic inflammatory disorder. Interestingly, we detected an accumulation of Caspase-8 in Atg5-deficient Treg cells. Since TCR restimulation of activated T cells can lead to activation-induced cell death through cell death receptors (48), it is tempting to speculate that the death receptor pathway contributes to Treg cell depletion and *Atg5* ^ΔFoxp3^ mouse phenotype.

Butyrate, a short chain fatty acids (SCFA) derived from the gut microbiota, facilitates the generation of intestinal Treg cells, mainly of RORγt^+^ Foxp3^+^ Treg cells (13, 49–51). Mitochondrial lipid oxidation, in part regulated by SCFA (52), promotes Treg cell proliferation *via* activation of the mTOR inhibitor AMP-activated kinase (53). Furthermore, inhibition of glycolysis favors Treg cell induction (54, 55). It has been shown that autophagy deficiency upregulates mTORC1 and c-Myc expression as well as activates the glycolytic pathway (18, 19). Such alterations cause metabolic programming dysregulation in Treg cells, having a direct impact on their ability to process microbiota-derived metabolites. Moreover, spermidine, a polyamine produced by commensal bacteria, restricts mTOR activity and enhances autophagy, potentiating Treg cell differentiation. Indeed, spermidine shifted Th17 *in vitro* differentiation towards Treg cells and ameliorated induced colitis in mice (56). All these data indicate that microbiota-derived metabolites are essential for intestinal Treg cell, especially RORγt^+^ Foxp3^+^ pTreg activity in an autophagy-dependent manner, giving a feasible explanation for the prominent intestinal pathology developed in *Atg5* ^ΔFoxp3^ mice.

Besides controlling intestinal inflammation, due to their immunosuppressive abilities, Treg cells can favor tumor growth. A recent study reported that RORγt^+^ Foxp3^+^ Treg cells sustain growth of colitis-associated colorectal cancer (57). Moreover, autophagy-dependent Treg stability and function benefits tumor progression (19, 58). Our findings show that the intestinal RORγt^+^ Foxp3^+^ Treg cell population depends on an intact autophagy machinery, which represent a potential target to enhance Treg cell activity in order to control intestinal inflammation or to restrict Treg cell-mediated tumor tolerance.

## Data Availability Statement

The raw data supporting the conclusions of this article will be made available by the authors, without undue reservation.

## Ethics Statement

The animal study was reviewed and approved by Niedersächsisches Landesamt für Verbraucherschutz und Lebensmittelsicherheit.

## Author Contributions

IS and TS designed and supervised the study. CP-S, BZ, AB, MP, NT, and MS performed the experiments and analyzed the data. CP-S and IS wrote the manuscript. All authors contributed to the article and approved the submitted version.

## Funding

This work was supported by grants of the Deutsche Forschungsgemeinschaft to I.S. (SCHM1586/3-1, SCHM1586/6-1), the Fritz Thyssen foundation to IS and the China Scholarship Council (CSC) to BZ (Grant #201704910936).

## Conflict of Interest

The authors declare that the research was conducted in the absence of any commercial or financial relationships that could be construed as a potential conflict of interest.

## Publisher’s Note

All claims expressed in this article are solely those of the authors and do not necessarily represent those of their affiliated organizations, or those of the publisher, the editors and the reviewers. Any product that may be evaluated in this article, or claim that may be made by its manufacturer, is not guaranteed or endorsed by the publisher.

## References

[1] Mizushima N Komatsu M. Autophagy: Renovation of Cells and Tissues. Cell (2011) 147:728–41. 10.1016/j.cell.2011.10.026 22078875

[2] Klionsky DJ Schulman BA. Dynamic Regulation of Macroautophagy by Distinctive Ubiquitin-Like Proteins. Nat Struct Mol Biol (2014) 21:336–45. 10.1038/nsmb.2787 PMC4036234 24699082

[3] Hanada T Noda NN Satomi Y Ichimura Y Fujioka Y Takao T. The Atg12-Atg5 Conjugate has a Novel E3-Like Activity for Protein Lipidation in Autophagy. J Biol Chem (2007) 282:37298–302. 10.1074/jbc.C700195200 17986448

[4] Weidberg H Shvets E Shpilka T Shimron F Shinder V Elazar Z. LC3 and GATE-16/GABARAP Subfamilies Are Both Essential Yet Act Differently in Autophagosome Biogenesis. EMBO J (2010) 29:1792–802. 10.1038/emboj.2010.74 PMC2885923 20418806

[5] Fontenot JD Gavin MA Rudensky AY. Foxp3 Programs the Development and Function of CD4+CD25+ Regulatory T Cells. Nat Immunol (2003) 4:330–6. 10.1038/ni904 12612578

[6] Hori S Nomura T Sakaguchi S. Control of Regulatory T Cell Development by the Transcription Factor Foxp3. Science (2003) 299:1057–61. 10.1126/science.1079490 12522256

[7] Vignali DAA Collison LW Workman CJ. How Regulatory T Cells Work. Nat Rev Immunol (2008) 8:523–32. 10.1038/nri2343 PMC2665249 18566595

[8] Bennett CL Christie J Ramsdell F Brunkow ME Ferguson PJ Whitesell L. The Immune Dysregulation, Polyendocrinopathy, Enteropathy, X-Linked Syndrome (IPEX) Is Caused by Mutations of FOXP3. Nat Genet (2001) 27:20–1. 10.1038/83713 11137993

[9] Brunkow ME Jeffery EW Hjerrild KA Paeper B Clark LB Yasayko SA. Disruption of a New Forkhead/Winged-Helix Protein, Scurfin, Results in the Fatal Lymphoproliferative Disorder of the Scurfy Mouse. Nat Genet (2001) 27:68–73. 10.1038/83784 11138001

[10] Wildin RS Ramsdell F Peake J Faravelli F Casanova JL Buist N. X-Linked Neonatal Diabetes Mellitus, Enteropathy and Endocrinopathy Syndrome Is the Human Equivalent of Mouse Scurfy. Nat Genet (2001) 27:18–20. 10.1038/83707 11137992

[11] Zhu J. T Helper Cell Differentiation, Heterogeneity, and Plasticity. Cold Spring Harb Perspect Biol (2018) 10:1–18. 10.1101/cshperspect.a030338 PMC6169815 28847903

[12] Lochner M Peduto L Cherrier M Sawa S Langa F Varona R. *In Vivo* Equilibrium of Proinflammatory IL-17+ and Regulatory IL-10+ Foxp3+ Rorγt+ T Cells. J Exp Med (2008) 205:1381–93. 10.1084/jem.20080034 PMC2413035 18504307

[13] Ohnmacht C Park JH Cording S Wing JB Atarashi K Obata Y. The Microbiota Regulates Type 2 Immunity Through Rorγt+ T Cells. Sci (80-) (2015) 349:989–93. 10.1126/science.aac4263 26160380

[14] Sefik E Geva-Zatorsky N Oh S Konnikova L Zemmour D McGuire AM. Individual Intestinal Symbionts Induce a Distinct Population of Rorγ^+^ Regulatory T Cells. Science (2015) 349:993–7. 10.1126/science.aaa9420 PMC4700932 26272906

[15] Yang BH Hagemann S Mamareli P Lauer U Hoffmann U Beckstette M. Foxp3+ T Cells Expressing Rorγt Represent a Stable Regulatory T-Cell Effector Lineage With Enhanced Suppressive Capacity During Intestinal Inflammation. Mucosal Immunol (2016) 9:444–57. 10.1038/mi.2015.74 26307665

[16] Jacquin E Apetoh L. Cell-Intrinsic Roles for Autophagy in Modulating CD4 T Cell Functions. Front Immunol (2018) 9:1023. 10.3389/fimmu.2018.01023 29867990 PMC5954027

[17] Le Texier L Lineburg KE Cao B McDonald-Hyman C Leveque-El Mouttie L Nicholls J. Autophagy-Dependent Regulatory T Cells Are Critical for the Control of Graft-*Versus*-Host Disease. JCI Insight (2016) 1:1–17. 10.1172/jci.insight.86850 PMC5033749 27699243

[18] Kabat AM Harrison OJ Riffelmacher T Moghaddam AE Pearson CF Laing A. The Autophagy Gene Atg16l1 Differentially Regulates Treg and TH2 Cells to Control Intestinal Inflammation. Elife (2016) 5:1–29. 10.7554/eLife.12444 PMC4798959 26910010

[19] Wei J Long L Yang K Guy C Shrestha S Chen Z. Autophagy Enforces Functional Integrity of Regulatory T Cells by Coupling Environmental Cues and Metabolic Homeostasis. Nat Immunol (2016) 17:277–86. 10.1038/ni.3365 PMC4755832 26808230

[20] Hara T Nakamura K Matsui M Yamamoto A Nakahara Y Suzuki-Migishima R. Suppression of Basal Autophagy in Neural Cells Causes Neurodegenerative Disease in Mice. Nature (2006) 441:885–9. 10.1038/nature04724 16625204

[21] Rubtsov YP Rasmussen JP Chi EY Fontenot J Castelli L Ye X. Regulatory T Cell-Derived Interleukin-10 Limits Inflammation at Environmental Interfaces. Immunity (2008) 28:546–58. 10.1016/j.immuni.2008.02.017 18387831

[22] Mizushima N Yamamoto A Matsui M Yoshimori T Ohsumi Y. *In Vivo* Analysis of Autophagy in Response to Nutrient Starvation Using Transgenic Mice Expressing a Fluorescent Autophagosome Marker. Mol Biol Cell (2004) 15:1101–11. 10.1091/mbc.e03-09-0704 PMC363084 14699058

[23] Stehr M Greweling MC Tischer S Singh M Blöcker H Monner DA. Charles River Altered Schaedler Flora (CRASF) Remained Stable for Four Years in a Mouse Colony Housed in Individually Ventilated Cages. Lab Anim (2009) 43:362–70. 10.1258/la.2009.0080075 19535393

[24] Lahl K Loddenkemper C Drouin C Freyer J Arnason J Eberl G. Selective Depletion of Foxp3+ Regulatory T Cells Induces a Scurfy-Like Disease. J Exp Med (2007) 204:57–63. 10.1084/jem.20061852 17200412 PMC2118432

[25] Turnbaugh PJ Ridaura VK Faith JJ Rey FE Knight R Gordon JI. The Effect of Diet on the Human Gut Microbiome: A Metagenomic Analysis in Humanized Gnotobiotic Mice. Sci Transl Med (2009) 1:6ra14. 10.1126/scitranslmed.3000322 PMC2894525 20368178

[26] Caporaso JG Lauber CL Walters WA Berg-Lyons D Lozupone CA Turnbaugh PJ. Global Patterns of 16S rRNA Diversity at a Depth of Millions of Sequences Per Sample. Proc Natl Acad Sci USA (2011) 108 Suppl:4516–22. 10.1073/pnas.1000080107 PMC3063599 20534432

[27] Caporaso JG Kuczynski J Stombaugh J Bittinger K Bushman FD Costello EK. QIIME Allows Analysis of High-Throughput Community Sequencing Data. Nat Methods (2010) 7:335–6. 10.1038/nmeth.f.303 PMC3156573 20383131

[28] Edgar RC. Search and Clustering Orders of Magnitude Faster Than BLAST. Bioinformatics (2010) 26:2460–1. 10.1093/bioinformatics/btq461 20709691

[29] Wang Q Garrity GM Tiedje JM Cole JR. Naive Bayesian Classifier for Rapid Assignment of rRNA Sequences Into the New Bacterial Taxonomy. Appl Environ Microbiol (2007) 73:5261–7. 10.1128/AEM.00062-07 PMC1950982 17586664

[30] McMurdie PJ Holmes S. Phyloseq: An R Package for Reproducible Interactive Analysis and Graphics of Microbiome Census Data. PloS One (2013) 8:e61217. 10.1371/journal.pone.0061217 23630581 PMC3632530

[31] Segata N Izard J Waldron L Gevers D Miropolsky L Garrett WS. Metagenomic Biomarker Discovery and Explanation. Genome Biol (2011) 12:R60. 10.1186/gb-2011-12-6-r60 21702898 PMC3218848

[32] Chassaing B Srinivasan G Delgado MA Young AN Gewirtz AT Vijay-Kumar M. Fecal Lipocalin 2, a Sensitive and Broadly Dynamic Non-Invasive Biomarker for Intestinal Inflammation. PloS One (2012) 7:e44328. 10.1371/journal.pone.0044328 22957064 PMC3434182

[33] Loos B du Toit A Hofmeyr J-HS. Defining and Measuring Autophagosome Flux—Concept and Reality. Autophagy (2014) 10:2087–96. 10.4161/15548627.2014.973338 PMC4502790 25484088

[34] Tanida I Ueno T Kominami E. LC3 and Autophagy. Methods Mol Biol (2008) 445:77–88. 10.1007/978-1-59745-157-4_4 18425443

[35] Walczak M Martens S. Dissecting the Role of the Atg12-Atg5-Atg16 Complex During Autophagosome Formation. Autophagy (2013) 9:424–5. 10.4161/auto.22931 PMC3590266 23321721

[36] Liston A Gray DHD. Homeostatic Control of Regulatory T Cell Diversity. Nat Rev Immunol (2014) 14:154–65. 10.1038/nri3605 24481337

[37] Pierson W Cauwe B Policheni A Schlenner SM Franckaert D Berges J. Antiapoptotic Mcl-1 Is Critical for the Survival and Niche-Filling Capacity of Foxp3^+^ Regulatory T Cells. Nat Immunol (2013) 14:959–65. 10.1038/ni.2649 PMC4128388 23852275

[38] Plaza-Sirvent C Schuster M Neumann Y Heise U Pils MC Schulze-Osthoff K. C-FLIP Expression in Foxp3-Expressing Cells Is Essential for Survival of Regulatory T Cells and Prevention of Autoimmunity. Cell Rep (2017) 18:12–22. 10.1016/j.celrep.2016.12.022 28052242

[39] Xu M Pokrovskii M Ding Y Yi R Au C Harrison OJ. C-MAF-Dependent Regulatory T Cells Mediate Immunological Tolerance to a Gut Pathobiont. Nature (2018) 554:373–7. 10.1038/nature25500 PMC5814346 29414937

[40] Griseri T Arnold IC Pearson C Krausgruber T Schiering C Franchini F. Granulocyte Macrophage Colony-Stimulating Factor-Activated Eosinophils Promote Interleukin-23 Driven Chronic Colitis. Immunity (2015) 43:187–99. 10.1016/j.immuni.2015.07.008 PMC4518500 26200014

[41] Maloy KJ Powrie F. Intestinal Homeostasis and Its Breakdown in Inflammatory Bowel Disease. Nature (2011) 474:298–306. 10.1038/nature10208 21677746

[42] Swidsinski A Ladhoff A Pernthaler A Swidsinski S Loening-Baucke V Ortner M. Mucosal Flora in Inflammatory Bowel Disease. Gastroenterology (2002) 122:44–54. 10.1053/gast.2002.30294 11781279

[43] Cosovanu C Neumann C. The Many Functions of Foxp3+ Regulatory T Cells in the Intestine. Front Immunol (2020) 11:600973. 10.3389/fimmu.2020.600973 33193456 PMC7606913

[44] Brandtzaeg P. Food Allergy: Separating the Science From the Mythology. Nat Rev Gastroenterol Hepatol (2010) 7:380–400. 10.1038/nrgastro.2010.80 20606633

[45] Kim BS Lu H Ichiyama K Chen X Zhang YB Mistry NA. Generation of Rorγt+ Antigen-Specific T Regulatory 17 Cells From Foxp3+ Precursors in Autoimmunity. Cell Rep (2017) 21:195–207. 10.1016/j.celrep.2017.09.021 28978473 PMC5716359

[46] Lathrop SK Bloom SM Rao SM Nutsch K Lio CW Santacruz N. Peripheral Education of the Immune System by Colonic Commensal Microbiota. Nature (2011) 478:250–4. 10.1038/nature10434 PMC3192908 21937990

[47] Josefowicz SZ Niec RE Kim HY Treuting P Chinen T Zheng Y. Extrathymically Generated Regulatory T Cells Control Mucosal TH2 Inflammation. Nature (2012) 482:395–9. 10.1038/nature10772 PMC3485072 22318520

[48] Krammer PH Arnold R Lavrik IN. Life and Death in Peripheral T Cells. Nat Rev Immunol (2007) 7:532–42. 10.1038/nri2115 17589543

[49] Arpaia N Campbell C Fan X Dikiy S van der Veeken J DeRoos P. Metabolites Produced by Commensal Bacteria Promote Peripheral Regulatory T-Cell Generation. Nature (2013) 504:451–5. 10.1038/nature12726 PMC3869884 24226773

[50] Furusawa Y Obata Y Fukuda S Endo TA Nakato G Takahashi D. Commensal Microbe-Derived Butyrate Induces the Differentiation of Colonic Regulatory T Cells. Nature (2013) 504:446–50. 10.1038/nature12721 24226770

[51] Smith PM Howitt MR Panikov N Michaud M Gallini CA Bohlooly-YM. The Microbial Metabolites, Short-Chain Fatty Acids, Regulate Colonic Treg Cell Homeostasis. Science (2013) 341:569–73. 10.1126/science.1241165 PMC3807819 23828891

[52] Schönfeld P Wojtczak L. Short- and Medium-Chain Fatty Acids in Energy Metabolism: The Cellular Perspective. J Lipid Res (2016) 57:943–54. 10.1194/jlr.R067629 PMC4878196 27080715

[53] Michalek RD Gerriets VA Jacobs SR Macintyre AN MacIver NJ Mason EF. Cutting Edge: Distinct Glycolytic and Lipid Oxidative Metabolic Programs Are Essential for Effector and Regulatory CD4+ T Cell Subsets. J Immunol (2011) 186:3299–303. 10.4049/jimmunol.1003613 PMC3198034 21317389

[54] Dang EV Barbi J Yang H Jinasena D Yu H Zheng Y. Control of T(H)17/T(reg) Balance by Hypoxia-Inducible Factor 1. Cell (2011) 146:772–84. 10.1016/j.cell.2011.07.033 PMC3387678 21871655

[55] Shi LZ Wang R Huang G Vogel P Neale G Green DR. HIF1α-Dependent Glycolytic Pathway Orchestrates a Metabolic Checkpoint for the Differentiation of TH17 and Treg Cells. J Exp Med (2011) 208:1367–76. 10.1084/jem.20110278 PMC3135370 21708926

[56] Carriche GM Almeida L Stüve P Velasquez L Dhillon-LaBrooy A Roy U. Regulating T-Cell Differentiation Through the Polyamine Spermidine. J Allergy Clin Immunol (2021) 147(1):335–48.e11. 10.1016/j.jaci.2020.04.037 32407834

[57] Rizzo A Giovangiulio M Stolfi C Franze E Fehling HJ Carsetti R. RORGT-Expressing Tregs Drive the Growth of Colitis-Associated Colorectal Cancer by Controlling IL6 in Dendritic Cells. Cancer Immunol Res (2018) 6:1082–92. 10.1158/2326-6066.CIR-17-0698 29991500

[58] Poillet-Perez L Sharp DW Yang Y Laddha SV Ibrahim M Bommareddy PK. Autophagy Promotes Growth of Tumors With High Mutational Burden by Inhibiting a T-Cell Immune Response. Nat Cancer (2020) 1:923–34. 10.1038/s43018-020-00110-7 PMC8409526 34476408

