# Supplementary material for: A Central Role for Atg5 in Microbiota-Dependent Foxp3+ RORγt+ Treg Cell Preservation to Maintain Intestinal Immune Homeostasis
Source: Front Immunol. 2021 Aug 26;12:705436. doi: 10.3389/fimmu.2021.705436 (PMC8427596; doi:10.3389/fimmu.2021.705436)
Supplement: Supplementary file 1 [file DataSheet_1.pdf]

Supplementary Material

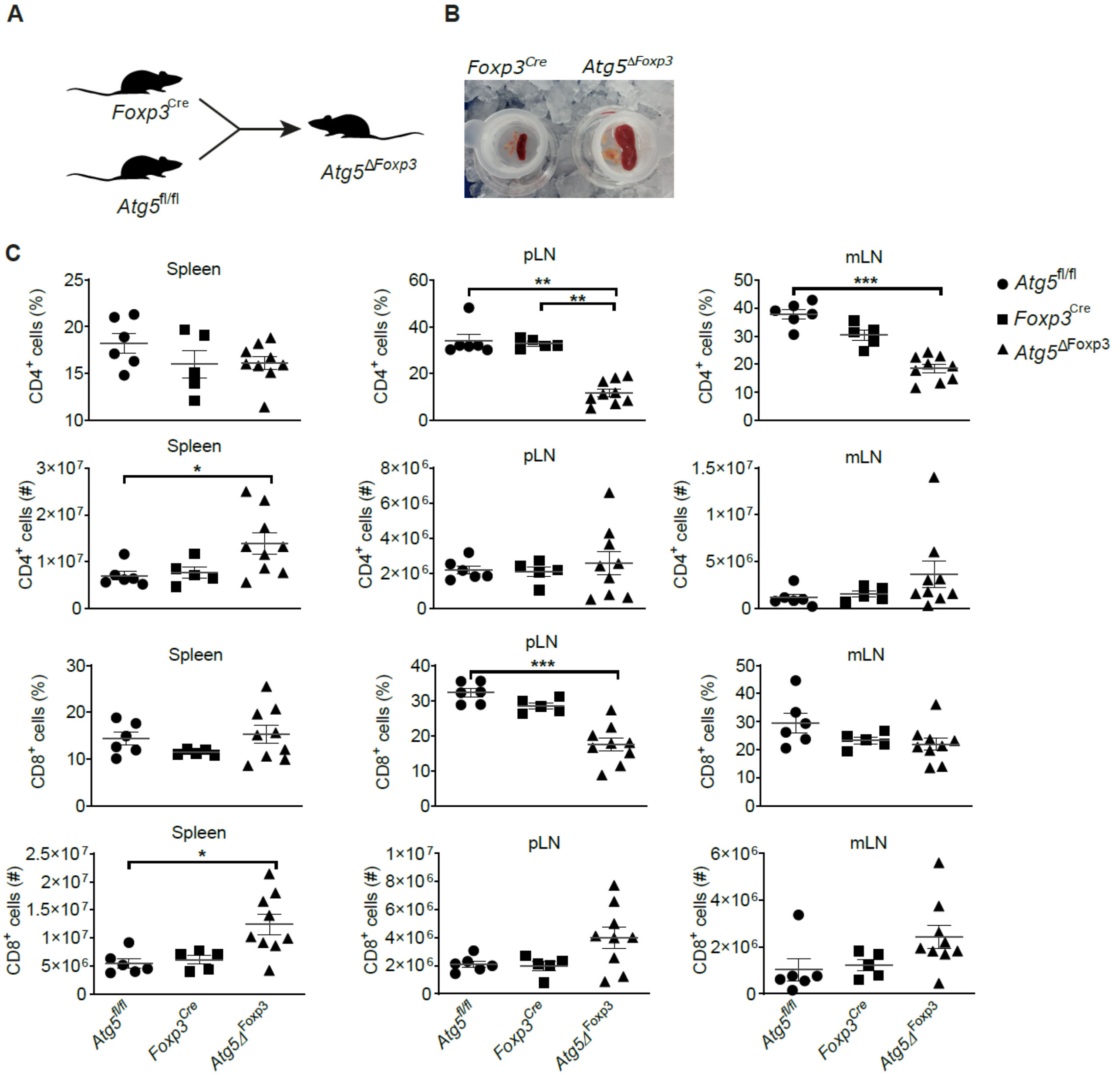

Supplementary Figure 1. **(A)** Schematic cartoon depicting the generation of the conditional knock out mouse *Atg5*$^{\Delta\text{Foxp3}}$ from the *Atg5*$^{fl/fl}$ and *Foxp3*^{Cre} mice. **(B)** Picture of spleen and lymph nodes from *Foxp3*^{Cre} and *Atg5*$^{\Delta\text{Foxp3}}$ mice. **(C)** CD4^{+} and CD8^{+} cell numbers and frequencies of spleen, peripheral lymph (pLN) and mesenteric lymph nodes (mLN) from 11-20 week old *Atg5*$^{fl/fl}$, *Foxp3*^{Cre} and *Atg5*$^{\Delta\text{Foxp3}}$ mice. The Kruskal–Wallis one-way ANOVA tests were used for statistical analysis. Mean ± SEM are given. Each symbol represents a single mouse in the scatter plots.

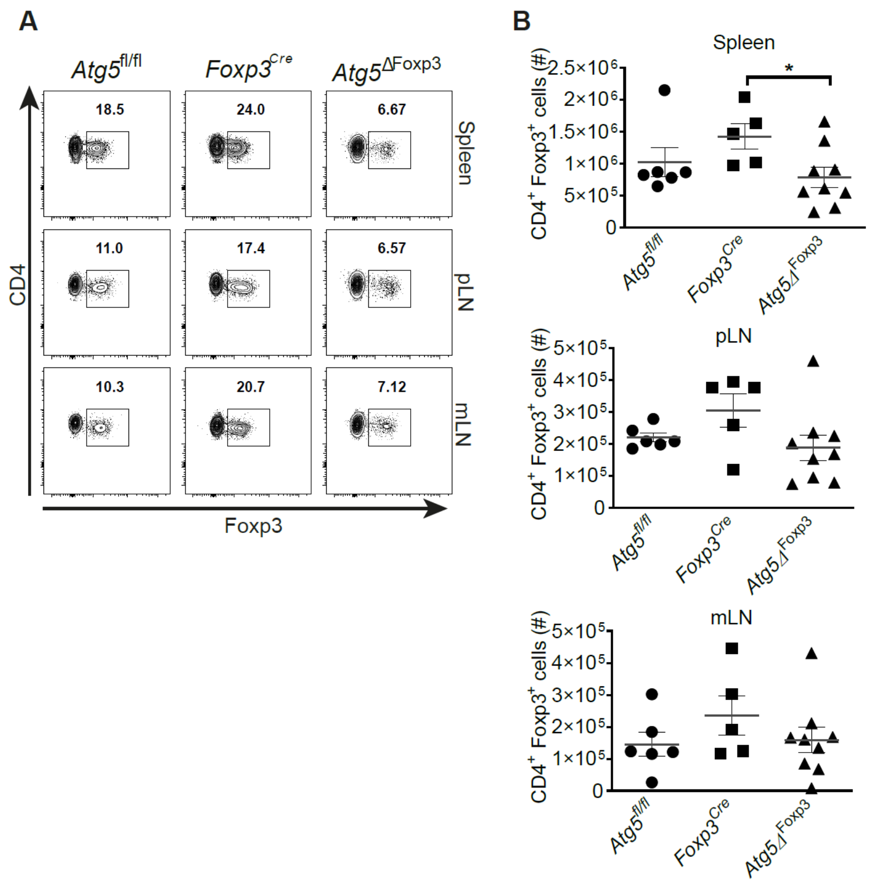

Supplementary Figure 2. (A) Representative contour plots of CD4^{+} Foxp3^{+} cell frequencies of spleen, peripheral lymph nodes (pLN) and mesenteric lymph nodes (mLN) from 11-20-week-old *Atg5$^{fl/f}$*, *Foxp3^{Cre}* and *Atg5*$^{\Delta Foxp3}$ mice. **(B)** Cell numbers of CD4^{+} Foxp3^{+} cells of spleen, peripheral lymph node (pLN) and mesenteric lymph node (mLN) from 11-20-week-old *Atg5$^{fl/f}$*, *Foxp3^{Cre}* and *Atg5*$^{\Delta Foxp3}$ mice. Mean ± SEM are given. Each symbol represents a single mouse in scatter plots. The Kruskal–Wallis one-way ANOVA tests were used for statistical analysis.

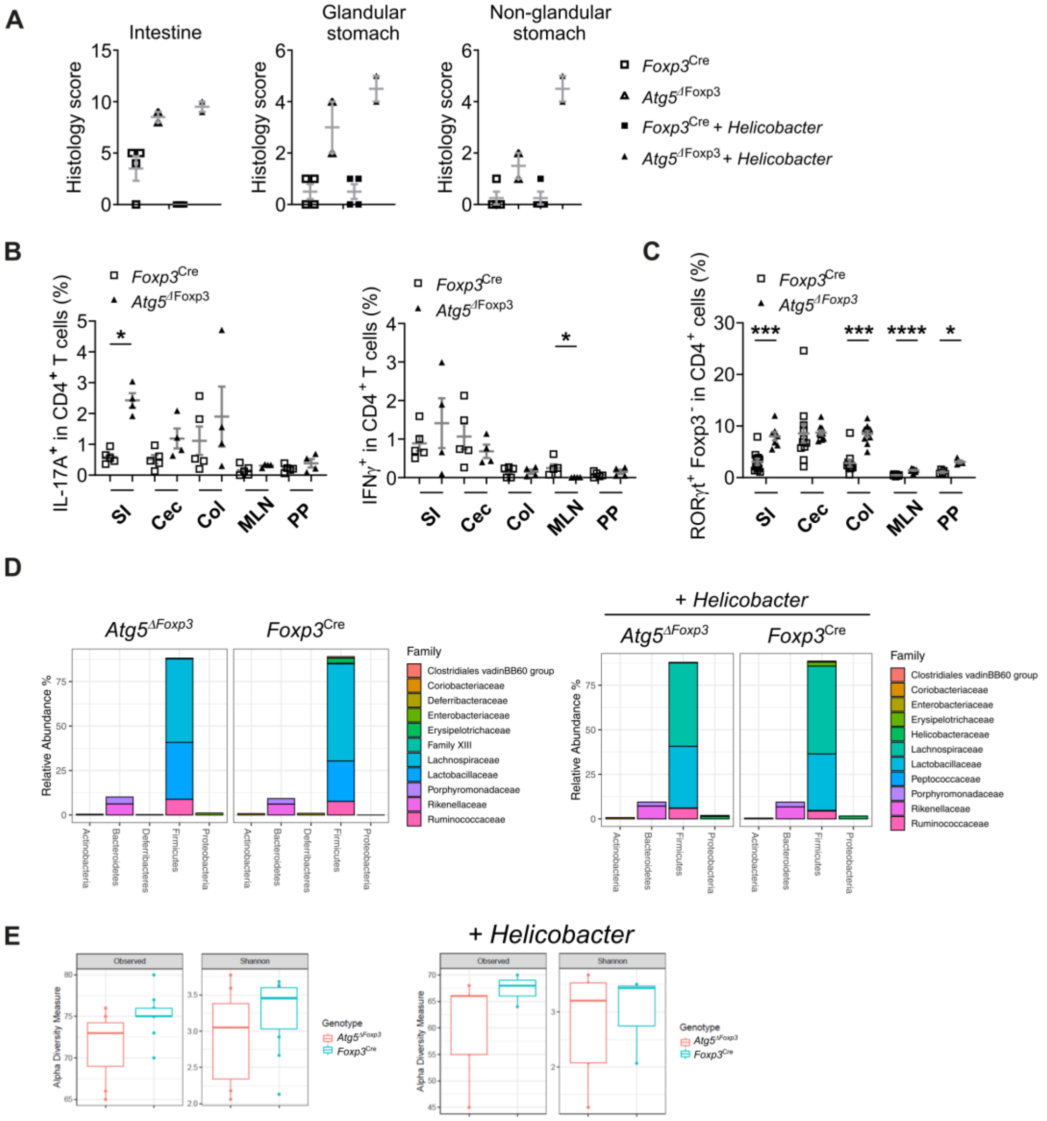

Supplementary Figure 3. (A) Histological score of stomach and intestinal injury in *Foxp3*^{Cre} and *Atg5*$^{\Delta Foxp3}$ mice under specific-pathogen-free conditions and colonized with Helicobacter species. **(B)** Frequency of IL-17A^{+} (left) and IFNγ^{+} cells (right) and **(C)** frequencies of RORγt^{+} Foxp3^{-} cells within CD4^{+} cells in small intestine (SI), cecum (Cec), colon (Col), mesenteric lymph nodes (MLN) and Peyer´s patches (PP) of *Foxp3*^{Cre} and *Atg5*$^{\Delta Foxp3}$ mice. **(D)** Representation of relative abundances of bacterial families and **(E)** alpha diversity and Shannon diversity index analysis of fecal microbiota from *Foxp3*^{Cre} and *Atg5*$^{\Delta Foxp3}$ mice under specific-pathogen-free conditions (left) and colonized with Helicobacter (right) using 16S rRNA sequencing. **(B, C)** Statistical analyses were performed by two-tailed Mann-Whitney tests. **(A-C)** Mean ± SEM are given. Each symbol represents a single mouse in the scatter plots.

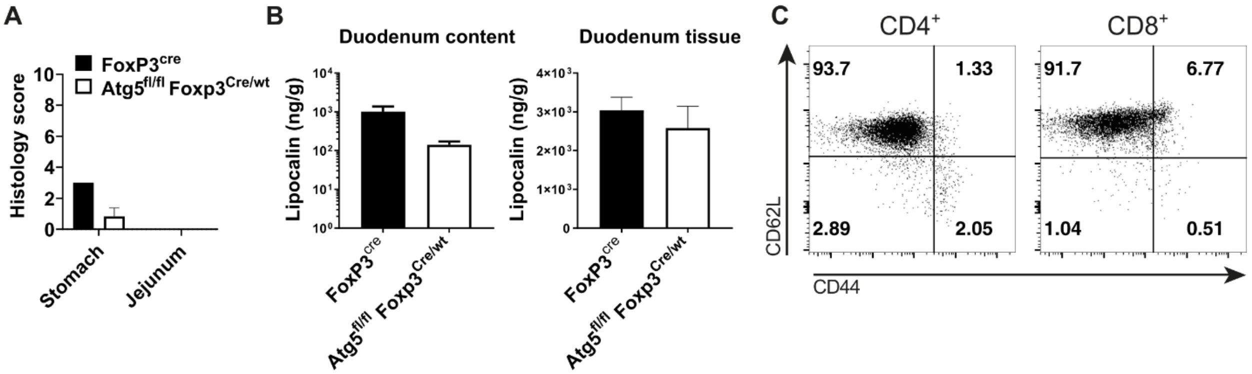

Supplementary Figure 4. (A) Histological score of stomach and jejunum inflammation in *Foxp3*^{Cre} and *Atg5*$^{fl/fl}$ *Foxp3*$^{Cre/wt}$ mice. **(B)** Lipocalin-2 levels in duodenum content and duodenum tissue of *Foxp3*^{Cre} (n=2) and *Atg5*$^{fl/fl}$ *Foxp3*$^{Cre/wt}$ mice (n=6). **(C)** Representative dot plots of T cell activation markers CD44 and CD62L in $CD4^+$ $Foxp3^-$ and $CD8^+$ cells from peripheral lymph nodes of 14 week-old *Atg5*$^{fl/fl}$ *Foxp3*$^{Cre/wt}$ mice (n=6).
